# The Importance of Ethnicity: Developing a Measure of Minority Ethnic Value and Value-Expressive Behavior Among Chinese Ethnic Minorities

**DOI:** 10.3389/fpsyg.2019.02603

**Published:** 2019-11-19

**Authors:** Yanli Yang, Fangmei Liang, Fangying Quan, Guangyu Jiang, Ke Yu, Yong Zheng

**Affiliations:** ^1^Center for Studies of Education and Psychology of Ethnic Minorities in Southwest China, Southwest University, Chongqing, China; ^2^Teaching Department of Public Course, Ningxia Art Vocational College, Yinchuan, China; ^3^Faculty of Psychology, Southwest University, Chongqing, China

**Keywords:** way of ethnicity, ethnic-minority-value, ethnic value-expressive behavior, validity, ethnic value-behavior relations

## Abstract

In this study we developed the Chinese Minority Ethnic Value Questionnaire (CMEVQ) and the Chinese Minority Ethnic Value-Expressive Behavior Questionnaire (CMEVEBQ) to assess the importance of ethnicity from the standpoint of diverse ethnic values and behavioral manifestations. Drawing on self-construal theory, social identity theory, and value theory, we conducted a review of literature, in-depth interviews, semi-structured questionnaires, and expert reviews. A total of 18 items for the CMEVQ and CMEVEBQ were developed. Data were collected from three samples of Chinese ethnic minorities (mainly college students). We generated two sets of item pools from the pilot sample (*n* = 438). Then we examined the dimensions and final items of the CMEVQ and CMEVEBQ using exploratory factor analysis (EFA) with sample 1 (*n* = 665). After that, we conducted a confirmatory factor analysis (CFA) to recheck the factor structure of two refined, mutually matched, yet independent scales obtained from Study 1 with sample 2 (*n* = 1309); meanwhile criterion-related, K-means cluster, *t*-tests, and multiple regression analyses were used to test the validity of and relationship between the CMEVQ and CMEVEBQ. Results showed that the multidimensional constructs with six shared first-order factors (Minority Ethnic Consciousness, Exploration, Involvement, Alienation, Inheritance, and Mastery) demonstrated a better fit for the data and supported the conceptual framework. Both questionnaires demonstrated adequate internal consistency reliability, convergent and discriminant validity, and ecological validity. That is, as practical, psychometrically sound measures, the CMEVQ and CMEVEBQ can be used to measure the importance of ethnicity for Chinese ethnic minorities. They also extend the content and sample fields of value research.

## Introduction

China is known to be a multiethnic nation, with the majority Han ethnic group (*Minzu*) representing mainstream culture and the 55 officially defined minority ethnic groups each with their own unique cultures. According to the famous sociologist and anthropologist Fei (1999), the term *minzu* refers to the unity of all ethnic groups in China, namely the whole Chinese nation, as well as to each individual ethnic group that makes up the Chinese nation. With the development of economic globalization, ethnic minorities who were once living in relatively closed environments have increased contact with modern mainstream culture, and the uniqueness of each ethnic minority has become increasingly challenged. Hence, maintaining the diversity of the Chinese nation and the ethnicity of minority ethnic members has become a pressing concern.

Verkuyten (2005, p. 198) contended that members of majority and minority ethnic groups can identify each other using four “ways of ethnicity”: being (individuals’ ethnic self-category; Phinney, 1996), feeling (individuals’ emotions or affect toward the ethnic group; Ong et al., 2010), knowing (individuals’ thoughts or cognition toward the ethnic group; Ong et al., 2010), and doing (individuals’ ethnic practices and social interaction; Phinney, 1992). However, the contents of each “way of ethnicity” that are particularly important or desirable in influencing ethnic members’ selection of actions and evaluation of events, remain undetermined. The goal of this study was to explore the “ways of ethnicity” of ethnic minority groups. Values are often important guidelines for people’s lives (Kluckhohn, 1951; Williams, 1968; Rokeach, 1973; Schwartz, 1992), and we thus we named these particularly important reflections of ethnicity “ethnic-minority-values.”

In fact, values tend to be defined at an abstract level in the real-world context, which makes it difficult to comprehend their content and importance; accordingly, the typically important ideals in measures of values are at least perceived by most individuals (Maio, 2010). When applied, values need to be related to something concrete, particularly to instantiations that can express them in ways other than subsuming diverse behaviors as exemplars of their concepts (Hanel et al., 2018). Researchers have gradually come to focus on value-expressive behaviors or to test the correlation between values and behavior in value-consistent directions (e.g., Verplanken and Holland, 2002; Schwartz and Butenko, 2014). Therefore, another aim of this study was to explore the value-expressive behaviors of ethnic minorities; that is, guided by ethnic-minority-values, it examined how ethnic members behaved to indicate their ethnicity and their relationships with their own ethnic groups. These behaviors may be driven either internally or externally, consciously or unconsciously, but should be chosen *a priori* to express their ethnic values (Schwartz and Butenko, 2014).

Drawing on self-construal theory, social identity theory (Tajfel, 1981; Tajfel and Turner, 1986), and value theory (Schwartz, 1992; Schwartz et al., 2012), we provided an operational definition for ethnic-minority-value, and developed two new mutually matched, yet independent, ethnic questionnaires, the Chinese Minority Ethnic Value Questionnaire (CMEVQ) and the Chinese Minority Ethnic Value-Expressive Behavior Questionnaire (CEMVEBQ). Inspired by Verkuyten’s (2005) ways of ethnicity and Phinney and Ong’s components of ethnic identity, we defined six shared dimensions for both questionnaires as follows: “being”—which we denoted as *minority ethnic consciousness* (MEC), “feeling”— *minority ethnic involvement* (MEIV) and *minority ethnic alienation* (MEA), “knowing”— *minority ethnic exploration* (MEE), and “doing”— *minority* ethnic inheritance (MEIH) and *minority ethnic mastery* (MEM). Multiple statistical methods were used to examine the psychometric properties, particularly the construct validity, of the two questionnaires.

### Ethnic-Minority-Values as a Necessary Part of Members’ Ethnic Self-Description: A Self-Construal Perspective

The emergence of a person’s various social identities is an essential prerequisite for the emergence of their personal identities and personality development (Swann and Bosson, 2010). Individuals’ initial self-construal is usually derived from the implicit or explicit expectations of various societal agents (e.g., family members) rather than beginning with self-awareness and self-reflection (Abrams, 2015). Tajfel and Turner (1986) defined social identity as “those aspects of an individual’s self-image that derive from the social categories to which he perceives himself as belonging” (p. 16). Thus, when an individual has an ethnic affiliation, their self-definition includes the expectations of their ethnic group, which has been found to be of considerable importance for self-concept among minority ethnic groups (Verkuyten, 2005). The “old” generation is unaware of the need to distinguish itself from others through intragroup comparison due to its common strong ethnicity. In contrast, the “new” generation, due to increasing contacts with “external” cultures, is more aware of intergroup and intragroup differences, which demonstrates their degree of acculturation and leads to wide variation in the recognition of importance of ethnicity. Fading cultural differences may cause members to emphasize their ethnic belonging, particularly for the most acculturated minority individuals (Verkuyten, 2005). Ethnicity may be of particular value in the self-construal of minority ethnic groups, which may influence choices and behaviors when activated (Verplanken et al., 2009).

### Ethnic-Minority-Value as a Basis of Ethnic Identity: A Social Identity Theory Perspective

Social identity theory (SIT: Tajfel and Turner, 1986) states that identity is linked to the value and evaluative significance of group membership. Ethnic identity, one part of social identity, incorporates both individuals’ knowledge of membership in their own ethnic group and the values and feelings attached to that membership (Tajfel, 1981, p. 255). Those who are characterized as high identifiers regard ethnic-group-value as being more important than those who are low identifiers (Verkuyten, 2006). Compared with the ethnic identity of the majority group, the ethnic identity of the minority group is not only related to its group but also to the “inside” of its own group. In other words, minority ethnic groups are “ethnic” from the “inside” due to a common imagined origin and culture, which are also used in constructing ethnic identity (Verkuyten, 2005). Further, ethnic cultures characterized by structure and continuation are usually expressed by factors such as central values and symbols. As previously stated, ethnic-minority-value is one factors that can identify an ethnic group. Thus, some researches on ethnic identity have not only considered values of an ethnic group as an essential component of ethnic identity, but also have used group-specific values as a variable to measure ethnic identity (e.g., Tajfel, 1981; Phinney and Ong, 2007).

### Ethnic Behavior as *a priori* Selection of the Value Expression of Ethnic Minority: A Value Theory Perspective

Since Verkuyten’s (2005) description of “doing” as one of the “ways of ethnicity,” ethnic behaviors, as ethnic practices and social interaction, have often been included in measures of ethnic identity. However, value theories have postulated that values are one significant factor of influence in everyday behavior and that behavior expresses important values to achieve the goals that are important to people and confirm the values that are critical to their self-identification (Schwartz, 2006). Furthermore, as the expression of underlying goal motivation (Roccas and Sagiv, 2010) and the mental representation of desirable goals (Maio, 2015), values encourage behaviors that promote and instantiate such goals. Here, we developed ethnic-minority-behavior as the *a priori* selection of the value expression of an ethnic minority. This was done for two main reasons: first, to instantiate the abstract concept of ethnic-minority-values; second, to preserve and confirm “ethnicity” from the standpoint of ethnic inheritance since “doing” or behaving is subject to change (Fishman, 1980).

In practice, however, behavior has proven particularly difficult to measure due to the variability caused by specific situational factors (Verkuyten, 2005). Multiple ethnic values may motivate most specific ethnic behaviors. For example, ethnic members may be motivated to participate in their own ethnic festivals by the values of ethnic involvement, ethnic consciousness, and/or ethnic inheritance. Furthermore, many ethnic behaviors express ethnic values more than they express other values. For example, spreading information on one’s own ethnic culture primarily expresses value in ethnic exploration while feeling cordial when observing matters related to one’s own minority group primarily expresses the value of ethnic involvement. Thus, we sought to explore whether each ethnic-minority -behavioral tendency can more typically express an ethnic-minority-value more than any other behavioral tendency, based on its conceptual definition. This required separate ethnic-minority-behavioral tendencies to be measured for each ethnic-minority-value. Additionally, for conceptual clarity, ethnic behaviors should be separated from ethnic values, which will allow the results to be measured and analyzed separately to distinguish the implications of value and its expressive behaviors.

### Definition of Ethnic-Minority-Value

The unique culture of each Chinese ethnic minority differentiates them from other minorities, which forms diverse eco-ethnological environments and sparks numerous relevant ethnological and sociological studies. Although there is no consensus on the concept of ethnic psychology in China, scholars generally have incorporated ethnic values as a basic element in ethnic psychology (e.g., Xu and Qi, 1996; Qi, 2000; Hou, 2008; Tu, 2010). Hou (2008) defines ethnic values as the sum of stable attitudes and choice tendencies, including feeling components, which come from evaluating the significance of ethnic culture by minority ethnic members according to their own needs. Tu (2010) has defined ethnic values as people’s most basic views on nationality and ethnic issues, which influence their attitudes and ways of dealing with specific ethnic issues, the essence of which is to safeguard and develop one’s own ethnic group’s interests and status. The term “*Minzu*” (ethnic group) in their definitions applies both to the majority ethnic group, such as ethnic Han, but also to minority ethnic groups. They did not exactly characterize the ethnicity of minority ethnic groups from the ethnic “inside.” Furthermore, several Western scholars have applied the term *ethnic value* as a psychometric indicator of immigrants’ acculturation (e.g., Koh et al., 2009) or ethnic identity (e.g., Phinney and Ong, 2007), but failed to explore its meaning. The present research attempted to enrich existing theory and methods of ethnic-minority-value using a sample of diverse and understudied minority ethnic groups in China.

Ethnic-minority-values have a complex, multidimensional structure encompassing various psychological traits of minority ethnic groups. As an essential component of ethnic identity, ethnic-minority-values refers to minority ethnic members’ self-perception of the importance of ethnicity based on their own ethnic contacts and practices; it indicates ethnic belonging, ethnic feeling, and recognition of one’s own ethnic group through, for example, ethnic language, customs, and religion, and is a subjective criterion for ethnic members to select and evaluate matters relevant to their own ethnic group. The concept of ethnic-minority-values is transforming. While some of its elements are gradually disappearing due to incompatibility with modern society, it also actively integrates new cultural factors, e.g., views, ways of life, or social norms (Hou, 2008). In addition, we have described ethnic-minority-value-expressive behaviors as a set of typical instantiations that express the content of various values among ethnic minorities.

### Overview of the Present Study

In summary, the present study aimed to (a) explore the content and construct validity of two new measures using experts’ reviews and diverse statistical procedures; (b) demonstrate support for two multidimensional measures that is consistent with the psychological operational definition of ethnic value among ethnic minorities in China; and (c) evaluate the criterion-related, discriminant, and ecological validity and relationships of the two new scales, which can be used collaboratively or independently. We purposefully sampled from multiple minority ethnic groups in China, regardless of demographic variables such as occupation and age, for two distinct benefits. First, the newly developed scales aimed to explore the common importance of ethnicity among minorities, and theoretically, they would be more directly affected by ethnicity strength than by demographic variables. Second, ethnic values and its corresponding behaviors could be affected by cultural context and minority ethnic living areas; thus, we sampled ethnic participants from a cultural context in which multiple minority groups live together to hold these influences constant.

## The Pilot Study

This study aimed to generate two sets of conceptually and empirically viable self-report item pools. Drawing on the format of item portrayal in Portrait Values Questionnaire (PVQ; Schwartz et al., 2012), items in CMEVQ described minority ethnic basic views on something important to their own ethnic groups, and CMEVEBQ used the act-frequency approach (Buss and Craik, 1983) to measure behavioral tendency to express minority ethnic values. All items are well-understood by the general population. We expected the item content in CMEVQ and CMEVEBQ to represent the importance of ethnicity by way of ethnic values and its postulated behavior tendency.

### Methods

#### Participants

Two samples were used that comprised 103 and 335 Chinese ethnic minority participants, respectively. Each participant’s minority ethnic identity was self-reported, and we assessed whether both participant and one of their parents were from the same ethnic group, which is the same method that was used in the subsequent studies reported in this paper. The first group was recruited from the ethnic department of Southwest University, and comprised participants educated to undergraduate level and above, including 86.4% (*n* = 89) students and 13.6% (*n* = 14) working people. Their mean age was 22.18 years (*SD* = 4.91), ranging from 17 to 40 years; 33.0% (*n* = 34) were men and 67.0% (*n* = 69) were women, 64.0% (*n* = 66) were from minority-inhabited areas, 36% (*n* = 37) were from minority-scattered areas, 67% (*n* = 69) were from rural areas, the parents of 60.2% (*n* = 62) of participants were of the same minority ethnic group, and 91.3% (*n* = 94) were single. Educationally, 78.6% (*n* = 81) were undergraduate students while 21.4% (*n* = 22) were graduate students. They included 20 of the 55 Chinese ethnic minorities: Miao, Tung, Tujia, Yi, Naxi, Gelao, Tibetan, Qiang, Mulam, Shui, Zhuang, Mongolian, Uygur, Hui, Yao, Buyi, Li, Dai, Kazakh, and Wa. Twelve participants agreed to participate in further interviews.

The second sample comprised 126 men and 209 women. They came from 12 of the 34 provinces/regions of China (based on IP address) and covered 26 Chinese ethnic minorities (Miao, Yi, Tujia, Bai, Tung, Hui, Buyi, Hani, Zhuang, Dai, Gelao, Uygur, Shui, Chuanqing, Tibetan, Mongolian, Manchu, Mulam, Naxi, Qiang, Yao, Korean, Kazakh, Lahu, Li, and Tu). Their ages ranged from 15 to 42 years with a mean age of 22.22 years (*SD* = 4.68). Professionally, there were 75.5% (*n* = 253) students and 24.5% (*n* = 82) working people; 64.5% (*n* = 216) were from minority-inhabited areas and 35.5% (*n* = 119) from minority-scattered areas; 47.2% (*n* = 158) were from rural areas, the parents of 75.8% (*n* = 254) of participants were of the same minority ethnic group, and 83.6% (*n* = 280) were single. Regarding educational level, 2% and 8.1% (*n* = 27) respectively had primary and junior middle school education, while 12.2% (*n* = 41) were senior high school students, 12.2% (*n* = 51) were junior college students, 58.8% (*n* = 197) were undergraduates, and 2.1% (*n* = 17) were graduate students.

#### Measures

The initial item pools of the CMEVQ and CMEVEBQ were generated following a semi-structured questionnaire, in-depth interviews with ethnic minorities, and existing questionnaires related to ethnic values (Hou, 2008; Tu, 2010; Hu et al., 2014) and ethnic behavior (Phinney, 1992; Phinney and Ong, 2007; Gaines et al., 2016). In addition to the demographic measures, the semi-structured questionnaires and interviews included the following open-ended questions: (1) Concerning minority ethnic consciousness: (a) Under what circumstances can you perceive your minority ethnic identity? (b) In your daily life, what actions have you ever engaged in to indicate your ethnic affiliation? (2) Regarding minority ethnic involvement: (a) What are your feelings for your own ethnic group? (b) How have you ever showed your feelings to your ethnic group in your life? (3) Concerning minority exploration: (a) How do you obtain knowledge about your ethnic group as you grow up? (b) What other ways do you think contribute to ethnic minorities’ exploring their own ethnic group? (4) Regarding minority ethnic mastery: (a) What do you think of the relationship between your ethnic group and society or nature? (b) What are the most important (most valuable) factors in making your ethnicity last longer? All initial questions were free of cultural content regarding specific minority ethnic groups to ensure they were applicable to any minority ethnic group.

Overall the initial item pools consisted of 63 items on the CMEVQ and 56 items on the CMEVEBQ. Each item in the CMEVQ comprised a short, gender-matched, verbal portrait of different minority ethnic member, each describing a *Minzu*-related goal, varying in importance to them and serving as guiding principles in maintaining their ethnicity. For example, “It is important to honor his/her own, diverse ethnic taboos.” The importance of this goal to an ethnic member suggests his/her ethnic consciousness and determines whether he/she will engage in relevant activities in his/her daily life whenever he/she has the opportunity. The respondents indicated how similar the ethnic member in the portrait is to themselves on a 6-point Likert-type response scale ranging from 1 (*not like me*) to 6 (*very much like me*) (Schwartz et al., 2012). The items in the CMEVEBQ were designed to measure how frequently the minority ethnic respondents had engaged in each *Minzu*-related behavior in their daily lives on a 4-point scale ranging from 0 (never doing) to 4 (always doing). The response “never had one opportunity to perform the behavior described by item” was labeled *X* and treated as missing data.

#### Procedure

The study was performed via an online Chinese survey platform^1^, which was used in the subsequent studies, using the snowball sampling method. To recruit the first group of participants, we sent the online link consisting of demographic characteristics and two questionnaires to minority ethnic undergraduates known to the authors, who then forwarded the link to their ethnic friends, classmates, and siblings. The respondents had 1 day to return the survey. The first group completed the semi-structured questionnaire, and 12 participants were then selected for in-depth interviews. Subsequently, the same researcher twice performed a content frequency analysis of similar and key information in 103 responses and 12 interviews, in terms of six populated pits – MEC, MEE, MEIV, MEA, MEIH, and MEM, over a 20-day interval. For the second group of participants we sent the online link, consisting of standard instructions explaining our research purpose, demographic variables, and the initial 63-item CMEVQ and 56-item CMEVEBQ based on the frequency analysis, to three teachers from Southwest Minzu University. They forwarded the online link to their class QQ or Wechat groups. The respondents were expected to individually complete the survey in the classroom within 20 min. As a monetary reward, we paid 10 Yuan RMB per survey submitted. We then conducted a statistical item-analysis of 335 responses to the two questionnaires and the experts reviewed the content validity and semantic analysis of the retained items.

#### Data Analyses

In the analysis of content frequency, specific ethnocultural matters (e.g., customs, rites, or taboos) were separately categorized as the frequency of common domains for all minority ethnic groups. For instance, Hui respondents mentioned Eid-al-Fitr, while Yi and Bai respondents mentioned the Torch festival or Dai’ Water-Splashing Festival, but the rater categorized all of them as festivals. Krippendorff’s alpha index was calculated to evaluate the intercoder reliability. Prior to item analysis, we replaced missing data, constituting less than 1% in both questionnaires, with a series mean. We adopted SPSS Version 22.0 for Windows (IBM Inc., Released 2013, Armonk, NY, United States) to operate the statistical item-analysis to delete items that had an item-total correlation coefficient of less than 0.30 and no significant difference at the *p* = 0.05 level between the highest and lowest 27% of the total population. Next, an ethnologist and psychology professor further evaluated those items retained in item-analysis.

### Results

Table 1 shows the information of frequency greater than 15 in the content frequency analysis of responses to the semi-structured questionnaires. Matters generally important to ethnic minorities comprise ethnic festivals, ethnic uniqueness, inheritance and development of ethnic culture, ethnic knowledge, ethnic language, ethnic customs, ethnic membership, and ethnic feeling. The Krippendorff’s nominal alpha presented a high degree of reliability of 0.87 based on the content frequency statistics of the rater, conducted twice (Krippendorff, 2007). Two initial item pools were shown as simplified Chinese statements, and some items were then eliminated because they did not meet the statistical criteria of item selection and experts’ review to item content. As a result, a total of 38 items were retained for the CMEVQ and 30 for the CMEVEBQ. Content assessment by statistical item-analysis and the experts supported our conceptualization of minority ethnic values and its behavioral tendency, indicating that they could be applied to the following studies.

**TABLE 1:** Frequency coding of Minority Ethnic Values and Value-Expressive Behaviors.

**Coding category**	**Value Matters/Behaviors**	***f***
Material Importance	Something exclusive to each ethnic minority (e.g., ethnic clothing, food, ornament, housing) Ethnic relics (e.g., traditional Miao Stockaded Village, Shui script, Potala Palace) Other *Minzu*-related information that can motivate ethnic consciousness (e.g., news reports, the word of the same to one’s own minority’s name, messages)	103 62 46
Mental and Spiritual Importance	Minority ethnic festivals (e.g., Eid-al-Fitr for Hui and Uygur, Memorial Ceremony for Mountain for Qiang, Outset Festival for Shui) Traditional ceremonies (e.g., sacrifices and religious activities, wedding, funeral) Inherit and develop the characteristic culture of one’s own minority (e.g., crafts, arts, education, economy, ecological environment) Learn about knowledge of one’s own ethnic minority (e.g., customs, origin, taboos, daily etiquettes) Minority ethnic language (e.g., Hmong language, Bai language, Bouyei language) and writing (e.g., Tai Lue, Yi characters, Tibetan Font) Deep feelings with ones’ own ethnic minority Intergenerational influences within the family Norms and moral customs of ones’ ethnic minority Religious belief (e.g. Benzhu worship for Bai, Nature and Totem worship, Dongba religion for Naxi)	103 97 91 85 76 37 35 26 19
Minority-related Behaviors	Obey customs (e.g., taboos, daily etiquettes, ceremonies) Show minority ethnic identity (e.g., self-introduction, choices with ethnic preference, native member gathering) Ways to explore one’s own ethnic knowledge (e.g., reading, ask for, talk about, minority activities) Pay attention to and forward *Minzu*-related information Support government’s measures to develop one’s own ethnic minority. Perception of inter-minority differences because of social context (e.g., living style, contact with other ethnic people) Integrate into ones’ own ethnic minority Mutual respect for folkways and customs (religious beliefs) of ethnic minorities Maintain interests of one’s own minority ethnic group (e.g., clarify misunderstanding, contributions Keep relationship with one’s own minority members	89 63 54 54 51 50 42 38 27 16

## Study 1

The aim of Study 1 was to explore the factor structure of the CMEVQ and CMEVEBQ using exploratory factor analysis (EFA), and to assess the internal reliability and construct validity of all latent factors with the structural equation model (SEM). We hypothesized that two multidimensional structures would fit the data better, indicating the need for two appropriate and parsimonious ethnic questionnaires, as well as several factors of ethnic-minority-values and value-expressive behavior.

### Method

#### Participants

We recruited another sample of 665 minority ethnic participants, comprising 235 (35.3%) women and 430 (64.7%) men from 19 provinces/regions of China (based on IP address) and covering 28 Chinese ethnic minorities (including all minority ethnic groups of the second group in the pilot study apart from ethnic Ge and Wa). Their ages ranged from 16 to 47 years, *M*_age_ = 22.26 years (*SD* = 4.90). Of these participants, 435 (65.4%) were from minority-inhabited areas; 503 (75.6%) were from rural areas, 107 (16.1%) were from towns, and 55 (8.3%) were from urban areas; 563 (84.7%) were unmarried; 530 (79.7%) were non-religious; and the parents of 502 (75.5%) participants were of the same minority ethnic group. Regarding educational level, 52 (7.8%) had received primary and junior middle education, 69 (10.4%) were senior high school students, 105 (15.8%) were junior college students, 398 (59.8%) were undergraduates, and 41 (6.2%) were graduate students. Professionally, there were 514 (77.2%) students and 151 (22.8%) working people. Our sample contained a broad range of demographics, which has been rarely involved in previous research.

#### Procedures

Data were collected from three schools: Tongren University in Guizhou Province, Southwest Minzu University in Sichuan, and Honghe University in Yunnan. These universities are located in minority ethnic areas and therefore have larger numbers of minority-registered students. The survey was conducted with the help of university administrators who forwarded an online link consisting of standard instructions explaining our research purpose, demographic variables, and two questionnaires to the class QQ or Wechat groups with a large number and variety of ethnic minorities. The respondents were expected to individually complete the survey in the classroom within 10 min. As a monetary reward, we paid 6 Yuan RMB per survey submitted. A total of 86 invalid questionnaires were eliminated, to give an effective survey rate of 88.7%.

#### Data Analyses

Prior to analysis, we replaced missing data, constituting less than 1% in both questionnaires, with a series mean. We used a principal component analysis with varimax rotation (Costello and Osborne, 2005) in SPSS 22.0 and parallel analysis in Mplus version 7 to examine the factor structure of the 38-item CMEVQ and 30-item CMEVEBQ by EFA. The following criteria of the “cleanest” factor structure were adopted: fixed number of six factors; item-loading on one factor above 0.40; no cross-loaded items; and no factors with more than three items. We only retained the three items with the highest factor loading for factors with more than three items (Hair et al., 2009). The SEM with maximum likelihood was performed to individually validate the possible psychometric models of the two questionnaires by IBM SPSS Amos version 22.0.

### Results

#### Factor Structure of the CMEVQ and CEMVEBQ

The results of the Kaiser-Meyer-Olkin (KMO) and Bartlett’s test of sphericity were KMO_CMEVQ_ = 0.948, χ^2^(435) = 8416.67 and KMO_CMEVEBQ_ = 0.932, χ^2^(435) = 5216.32, *p*_*s*_ < 0.001, which supported the factorial analysis. After the principal component analysis of each questionnaire, visual analysis of two scree plots individually suggested six “jumps” down to the scree line. Table 2 contains the first eight Eigenvalues of total variance of the retained CMEVQ and CMEVEBQ items. We conducted parallel analysis to extract the number of factors of the two scales, and the results separately suggested five and four factors for CMEVQ and CMEVEBQ, respectively. Thus, we conducted another principal component analysis of the two questionnaires; the items that did not meet the above item-selection criteria were gradually eliminated, specifying the number of factors as six. A value item stating “It is important to him/her to have more educational opportunities for children of their own minority” loaded 0.43 and 0.59 onto two factors, and we retained this item to the latter factor to ensure it has at least three items. Finally, a total of 18 items of the CMEVQ and CMEVEBQ with six-factor structures were generated, individually accounting for 66.79 and 63.67% of the total variance in the item correlation matrix. The factor loadings of the two questionnaires are displayed in Tables 3, 4, respectively. The Cronbach’s alpha coefficients for each factor with only three items suggested that the CMEVQ and CMEVEBQ are robust, except for the MEM factor. Reliability coefficients of the total scores were 0.759 for CMEVQ and 0.767 for CMEVEBQ. The fit indices of the value and behavior models are shown in line 1 of the two panels of Table 5, demonstrating that both CMEVQ and CMEVEBQ had reasonable construct validity with six first-order factors.

**TABLE 2:** Total variance explained for CMEVQ and CMEVEBQ.

**Component**	**Initial Eigenvalues**			**Extraction Sums of Squared Loadings**			**Rotation Sums of Squared Loadings**		
			
	**Total**	**% of Variance**	**Cumulative %**	**Total**	**% of Variance**	**Cumulative %**	**Total**	**% of Variance**	**Cumulative %**
V-1	10.59	35.29	35.29	10.59	35.29	35.29	5.87	19.55	19.55
V-2	1.75	5.83	41.12	1.75	5.83	41.12	3.94	13.15	32.70
V-3	1.54	5.13	46.25	1.54	5.13	46.25	2.86	9.52	42.22
V-4	1.30	4.32	50.57	1.30	4.32	50.57	1.83	6.09	48.31
V-5	1.06	3.52	54.09	1.06	3.52	54.09	1.38	4.59	52.91
V-6	0.98	3.25	57.34	0.98	3.25	57.34	1.33	4.43	57.34
V-7	0.93	3.11	60.45						
V-8	0.91	3.03	63.48						
B-1	9.61	32.02	32.02	9.61	32.02	32.02	4.68	15.59	15.59
B-2	1.87	6.22	38.24	1.87	6.22	38.24	3.60	12.01	27.61
B-3	1.58	5.28	43.52	1.58	5.28	43.52	2.83	9.42	37.03
B-4	1.25	4.18	47.70	1.25	4.18	47.70	2.18	7.28	44.31
B-5	1.19	3.96	51.65	1.19	3.96	51.65	2.07	6.89	51.20
B-6	1.12	3.73	55.39	1.12	3.73	55.39	1.26	4.19	55.39
B-7	0.98	3.27	58.66						
B-8	0.93	3.09	61.74						

*V and B refer to the factor in CMEVQ and CMEVEBQ, respectively.*

**TABLE 3:** First-order factor structure of the Chinese Minority Ethnic Value Questionnaire.

**Component and item description**	***M***	***SD***	**α**	**loading**	**C**	**E**	**V (%)**
MEC1 He/she agrees with the traditional norms of his/her own minority to deal with matters.	3.67	1.27	0.61 0.69^∗^	0.60	0.63	1.59	8.81
MEC2 It is important to him/her to honor his/her own ethnic diverse taboos.	4.26	1.28		0.64	0.66		
MEC3 It is important to him/her to form own minority consciousness due to cultural differences while in intercourse with different ethnic groups.	4.34	1.11		0.72	0.61		
MEE1 It is important to him/her to know more about his/her own ethnic group.	4.68	1.09	0.82 0.76^∗^	0.75	0.71	2.39	13.29
MEE2 It is important to him/her to get acquaintance with his/her ethnic group by oral transmission of ethnic legends and proverbs.	4.85	0.99		0.77	0.74		
MEE3 It is important to him/her to enhance ethnic consciousness by some ways (e.g., gathering with one’s own ethnic members, celebrating ethnic festivals).	4.68	0.99		0.72	0.69		
MEIV1 He/she believes that the sacrificial activities of his/her own minority can bring about well-being.	3.66	1.35	0.81 0.70^∗^	0.79	0.66	2.44	13.57
MEIV2 It is important to him/her to have a feeling of inseparability for his/her own minority.	4.18	1.25		0.86	0.78		
MEIV3 It is important to him/her to hold a ceremony in accordance with his/her own ethnic traditional customs (e.g., sacrifices, weddings and funerals).	4.57	1.17		0.77	0.73		
MEA1 It is not important to him/her whether minority members can administrate his/her ethnic internal affairs.	2.93	1.49	0.58 0.70^∗^	0.66	0.69	1.71	9.50
MEA2 It is not important to him/her to inherit and develop his/her own ethnic characterized culture.	2.25	1.41		0.75	0.65		
MEA3 It is not important to him/her whether he/she can do something for his/her own minority group or not.	2.48	1.49		0.73	0.58		
MEIH1 It is important to him/her that his/her ethnic cultural heritage can get preservation.	5.01	0.85	0.74 0.79^∗^	0.67	0.58	2.11	11.72
MEIH2 It is important to him/her that the state invest more to protect and inherit his/her ethnic characterized culture.	5.04	0.90		0.66	0.66		
MEIH3 It is important to him/her to inherit his/her own ethnic culture by some ways (e.g., ethnic festivals or written record).	4.86	1.02		0.56	0.62		
MEM1 It is important to him/her that ethnic minorities enjoy more preferential treatment from ethnic policy.	4.78	1.14	0.69 0.69^∗^	0.85	0.75	1.78	9.89
MEM2 It is important to him/her that his/her own minority ethnic interests would not be violated.	4.85	1.04		0.62	0.57		
MEM3 It is important to him/her that children of their own ethnic group have more educational opportunities.	5.16	0.89		0.59	0.70		

*^∗^Value denotes the internal consistency of the factor in CFA of study 2. α, Cronbach’s alpha coefficients; C, communality; E, Eigenvalue; V, Variance (%). MEC, Minority Ethnic Consciousness; MEE, Minority Ethnic Exploration; MEIV, Minority Ethnic Involvement; MEA, Minority Ethnic Alienation; MEIH, Minority Ethnic Inheritance; MEM, Minority Ethnic Mastery.*

**TABLE 4:** First-order factor structure of the Chinese Minority Ethnic Value-expressive Behavior Questionnaire.

**Component and item description**	***M***	***SD***	**α**	**loading**	**C**	**E**	**V**
MEC1 Celebrate your own ethnic festivals with families and friends.	1.96	1.23	0.69 0.66^∗^	0.73	0.63	1.72	9.58
MEC2 Consider the ethnic factors when making choices (e.g., schools, residential areas, spouse, elections, and the ethnic identity of descendants).	2.71	0.96		0.65	0.63		
MEC3 Attend actively interaction activities related to your own ethnic group.	2.90	1.00		0.56	0.69		
MEE1 Read about information that are related to your own ethnic group by wechat, microblog, or e-book.	2.21	0.96	0.73 0.75^∗^	0.74	0.63	2.23	12.40
MEE2 Discuss diverse ethnic topics (e.g., ethnic customs, ethnic development, ethnic education).	2.23	0.96		0.72	0.68		
MEE3 Spread information on your own ethnic culture.	2.44	0.98		0.63	0.58		
MEIV1 Feel regretful that your ethnic traditional cultures are slowly fading away.	2.97	0.94	0.62 0.65^∗^	0.78	0.52	1.81	10.07
MEIV2 Feel cordial when seeing matters (news, ethnic names, ethnic clothing, etc.) related to your ethnic group.	3.38	0.81		0.62	0.61		
MEIV3 Recommend the ethnic tourism projects-characterized in your hometown to others.	2.83	0.97		0.47	0.62		
MEA1 Not know and not seek the answers as well when asked about knowledge about your ethnic group.	0.71	0.97	0.69 0.69^∗^	0.78	0.65	2.01	11.15
MEA2 Pay no attention to your ethnic information.	1.17	0.94		0.75	0.67		
MEA3 Not care whether you still self-perceive your own ethnicity.	0.84	0.97		0.74	0.67		
MEIH1 Support various public welfare projects (free clinics, donations, poverty alleviation, etc.) in your ethnic settlement areas to enhance their vitality.	2.82	1.03	0.74 0.78^∗^	0.80	0.70	2.28	12.67
MEIH2 Support the construction of public facilities (e.g., kindergartens, community cultural centers, medical and health centers) in your ethnic settlement areas to enhance their vitality.	2.99	0.95		0.79	0.71		
MEIH3 Support a development of *Minzu*-characterized industries (local specialty, handicraft, etc.) in your ethnic settlement areas.	2.98	0.90		0.62	0.55		
MEM1 Objectively treat and deal with the disharmony between your own ethnic group members and other group members.	2.86	0.87	0.45 0.55^∗^	0.74	0.65	1.40	7.81
MEM2 Support natural resources from ethnic region where you inhabit to be allocated to use by the state.	2.99	0.88		0.58	0.67		
MEM3 Introduce some more convenient lifestyles (e.g., mobile payment, purchase, daily necessities) or interpret new national policies to the elders and adolescents of your own ethnic group.	2.57	1.01		0.55	0.67		

*^∗^Value denotes the internal consistency of the factor in CFA of study 2. α, Cronbach’s alpha coefficients; C, Communality; E, Eigenvalue; V, Variance (%). MEC, Minority Ethnic Consciousness; MEE, Minority Ethnic Exploration; MEIV, Minority Ethnic Involvement; MEA, Minority Ethnic Alienation; MEIH, Minority Ethnic Inheritance; MEM, Minority Ethnic Mastery.*

Following an inspection of the item contents of each facet, combined with Verkuyten’s four “ways of ethnicity” and other authors’ specific content descriptions of those ways, we defined the meaning of each postulated facet. MEC refers to minority ethnic self-awareness of own ethnic affiliation and of matters related to own ethnic group. MEE refers to the process of minority ethnic members seeking or being exposed to own ethnic group related knowledge. MEIV refers to the degree of ethnic minorities’ emotional cognition of own ethnic groups and ethnic affiliation. MEIH refers to the important aspects that can secure the longevity of a minority ethnic group. MEM refers to the relationship between an ethnic minority and society and nature. As a facet of the cognitive unimportance of ethnicity in the values and behavior questionnaire, MEA refers to the degree minority ethnic members’ self-perceived alienation from their own ethnic group and was assumed to be opposite to the other five facets from the standpoint of ethnicity.

The composite reliability was computed to test the internal consistency or suitability of each latent variable. In the CMEVQ and CMEVEBQ, MEC = 0.61 and 0.72, MEE = 0.82 and 0.74, MEIV = 0.82 and 0.69, MEA = 0.59 and 0.69, MEIH = 0.74 and 0.77, and MEM = 0.71 and 0.59. Most values exceeded the critical level of 0.60 and were above 0.70, while only two values approached 0.60, demonstrating reasonable internal fit indices for all latent factors (Bagozzi and Yi, 1988).

#### Measurement Invariance of Ethnic-Minority-Values and Value-Expressive Behaviors

In view of Verkuyter’s four “ways of ethnicity,” we also explored separate four-factor models for CMEVQ and CMEVEBQ, and we obtained the same items as with the six-factor model. Then we conducted an SEM, and the results of fit indices are presented in line 3 of the two panels of Table 5. By comparison, the fit indices of the two four-factor models were poorer than that of the six-factor models, but most were acceptable.

**TABLE 5:** Goodness-of-fit statistics for the confirmatory factor analysis of CMEVQ and CEMVEBQ: A summary.

**Scale**	**Model Description**	**N (*n*)**	***x*^2^**	***Δx^2^***	***df***	**Δdf**	***x*^2^*/df***	**CFI**	**GFI**	**AGFI**	**RMSEA [90% CI]**	**SRMR**	**ΔCFI**	**ΔTLI**
**CEMVQ**														
	Revised values model (6 factor)	665	353.73		120		2.948	0.943	0.945	0.921	0.054 [0.048, 0.061]	0.043		
	Revised values model (6 factor)	1309	447.16		95		4.707	0.966	0.960	0.935	0.053 [0.048, 0.058]	0.035		
	Revised values model (4 factor)	665	602.70		129		4.672	0.884	0.904	0.873	0.074 [0.068, 0.080]	0.065		
	Revised values model (4 factor)	1309	1184.8		113		10.485	0.897	0.892	0.853	0.085 [0.081, 0.090]	0.050		
Sample- related	Value model for samples from the inhabited areas	435	275.55		120		2.296	0.938	0.934	0.907	0.055 [0.046, 0.063]	0.046		
	Value model for samples from the scattered areas	230	247.50		120		2.063	0.918	0.899	0.856	0.068 [0.056, 0.080]	0.057		
MG-CFA	Factor loading invariance	435/230	546.99	23.81	252	12	2.171	0.927	0.918	0.889	0.042 [0.037, 0.047]	0.060	−0.003	−0.001
	Structural invariance	435/230	583.92	36.93	273	21	2.139	0.923	0.913	0.891	0.041 [0.037, 0.046]	0.071	−0.004	−0.002
**CEMVEBQ**														
	Revised behaviors model (6 factors)	665	244.75		120		2.040	0.960	0.956	0.937	0.042 [0.034, 0.049]	0.038		
	Revised behaviors model (6 factors)	1309	420.60		118		3.564	0.963	0.966	0.950	0.044 [0.040, 0.049]	0.032		
	Revised behaviors model (4 factors)	665	397.33		129		3.080	0.913	0.927	0.903	0.059 [0.052, 0.066]	0.047		
	Revised behaviors model (4 factors)	1309	639.30		129		4.956	0.938	0.947	0.929	0.055 [0.051, 0.059]	0.036		
Sample- related	Behaviors model for samples from the inhabited areas	435	254.54		120		2.121	0.931	0.938	0.911	0.051 [0.042, 0.059]	0.050		
	Behaviors model for samples from the scattered areas	230	145.56		120		1.213	0.977	0.938	0.912	0.031 [0.000, 0.047]	0.042		
MG-CFA	Factor loading invariance	435/230	408.76	8.64	252	12	1.622	0.949	0.936	0.914	0.031 [0.025, 0.036]	0.043	0.002	−0.005
	structural invariance	435/230	440.09	31.33	273	21	1.612	0.945	0.931	0.914	0.030 [0.025, 0.036]	0.052	−0.004	−0.001

**x*^2^, normal theory weighted least squares chi-square; *df*, degrees of freedom; RMSEA, root-mean square error of approximation; CFI, comparative fit index; GFI, goodness of fit index; AGFI, adjusted GFI; Δχ^2^, difference in χ^2^ values between models; Δdf, difference in number of degrees of freedom between models; ΔCFI, difference in CFI; ΔTLI, difference in Tucker-Lewis Index.*

Furthermore, a multiple-group comparison was conducted between the minority groups from the minority-inhabited areas and minority-scattered areas to test measurement invariance of the internal structure of ethnic-minority-values and behaviors models by the multiple-group confirmatory factor analysis (MG-CFA). Lines 2 and 3, in the top and bottom panels of Table 5 respectively, presented the fit indexes of sample models across settlement areas. The final two lines of the two panels respectively presented Goodness-of-fit results of invariant factor loadings and structural invariance. Although the χ^2^ from both the factor-loading and configural model of values were statistically significant [Δχ^2^_(__12__)_ = 23.808, *p* = 0.022; Δχ^2^_(__21__)_ = 36.927, *p* = 0.017], the differences between the CFI values met the recommended cutoff criterion of 0.01 (ΔCFI = −0.003 and −0.004; Cheung and Rensvold, 2002), and the differences between Tucker-Lewis Index (TLI) values also met the critical level of 0.5 (ΔTLI = −0.001 and −0.002; Little, 1997).

### Discussion

These results provided preliminary support for our view that ethnic-minority-values could be conceptualized and represented by ways of ethnicity. As expected, both initial questionnaire data indicated the multidimensional constructs that represented six correlated first-order specific factors. Although values are generally considered to comprise what is important to people in life, there appear to be distinct manifestations of ethnicity-related views and behaviors. The test results of measurement invariance provided evidences of two better-fitting models of the CMEVQ and CMEVEBQ; combining the conceptual contents of ethnic-minority-value and value-expressive behavior, both six-factor models were appropriate and parsimonious for use in Study 2. As for the lower internal consistency of the MEM value subscale, this may be related to small items or its poor domain representation, and requires further verification.

## Study 2

Study 2 aimed to check the internal consistency reliability of the newly developed CMEVQ and CMEVEBQ for Chinese multiethnic minorities, as well as to validate their construct, criterion-related, discriminant, and ecological validity. It was hypothesized that the CMEVQ and CMEVEBQ would demonstrate adequate psychometric properties and be useful in expressing ethnicity from the minority ethnic “inside.” Furthermore, much of the evidence on value-behavior associations has provided reason to believe that values have at least some causal influence on behavior. In particular, studies that value manipulations affect subsequent behaviors in value-consistent directions (e.g., Verplanken and Holland, 2002; Maio, 2015). This study did not assess causality, but did statistically test the relationship between ethnic values and their postulated behaviors. We expected that ethnic behaviors would be more strongly correlated with their related ethnic values than with other values.

### Method

#### Participants

In the final analyses, there were 1309 surveys covering 39 Chinese ethnic minorities (Lisu, Pumi, Blang, Dongxiang, Jingpo, Salar, She, Wa, Achang, Daur, Maonan, Nu, Tatar, and the 26 minorities of the second group in the pilot study) ranging in age from 15 to 26 years, *M*_age_ = 20.69 year (*SD* = 2.14). In the sample, 788 (60.2%) were women and 521 (39.8%) were men, 809 (61.8%) were from minority-inhabited areas, and 974 (74.4%) were non-religious. Regarding respondents’ homeplace, 548 (41.9%) were from rural areas, 383 (29.3%) were from towns, and 378 (28.9) were from urban areas. The parents of 61.5% (*n* = 805) of participants were of the same minority ethnic group, while those of 504 (38.5%) were of two ethnic groups, of which 468 were descendants of an ethnic minority and ethnic Han.

#### Design

Participants responded to a series of scales online: the CMEVQ and CMEVEBQ scales developed in Study 1; the Inclusion of Ingroup in the Self (IIS; Tropp and Wright, 2001); the Chinese Multiethnic Adolescent Cultural Identity Questionnaire (CMACIQ; Hu et al., 2014); the Ethnic Self-Categorization (ESC; Verkuyten and De Wolf, 2002); the Collective Self-Esteem Scale (CSE); and demographic questions.

#### Measures

The IIS consisted of seven Venn-like diagrams, with pairs of circles varying in their degree of overlap. As a single-item measure, the IIS proved to be a valid and reliable measure to assess ingroup identification. We used it to quickly measure the level of identification for ethnic minority members to their own minority ingroup. It was also particularly suitable for multi-minority ethnic group memberships. The word “group” here was labeled as “Your own ethnic group.”

The CMACIQ consisted of two higher-order factors, six first-order factors, and 34 items, and was rated on a 5-point scale ranging from 1 (*strongly disagree*) to 5 (*strongly agree*). We only used three of the first-order factors, Preference for Ethnic Things (e.g., “Prefer to eat your own ethnic traditional foods”), Ethnic Acceptance (e.g., “My ethnic group is honest and trustworthy”), and Social Norms (e.g., “Loyalty and filial piety are the Chinese nation’s fundamental values”). The Spearman-Brown split-half and test-retest correlations exceeded 0.70 over a 2-week interval. The internal reliability of the three facets used in this study were 0.83, 0.81, and 0.84, respectively.

The ESC had three items. In view of the sample’s identity in this study, the word “Chinese” in the original items was replaced and the revised contents read “I see myself as a typical member of my minority,” “I am very similar to other members of my minority in how I feel about things,” and “I think the same as other members of my minority about important things in life.” Responses were provided on a 5-point scale ranging from 1 (*complete disagreement*) to 7 (*complete agreement*). The internal reliability was high in this study, at 0.82.

The CSE was designed to assess the positivity of one’s social or collective identity. It included four subscales and 16 items on a 7-point scale ranging from 1 (*strongly disagree*) to 7 (*strongly agree*). Verkuyten and De Wolf (2002) use four items of the CSE’s private subscale to measure ethnic self-esteem (ESE). We adopted three subscales: Membership Esteem (four items, e.g., “I am a worthy member of the social groups I belong to”), in which the item “I am a cooperative participant in the social groups I belong to” was not included in our study due to inappropriate content, Private Collective Self-Esteem (four items, e.g., “I feel good about the social groups I belong to”), and Importance to Identity (four items, e.g., “The social groups I belong to are an important reflection of who I am”). In this study, the original term, “social group” referred to “minority ethnic group,” so we replaced this term with “minority ethnic group” (e.g., “I feel good about the minority ethnic group I belong to”). Reliability analyses revealed substantial alphas of their subscales ranging from 0.73 to 0.80. The three subscale alphas in this study were 0.62 (three items used), 0.70, and 0.75, respectively.

#### Procedures

To explore the characteristics of ethnic-minority-value and value-expressive behavior in multiethnic contexts, we mainly sampled indigenous ethnic minorities from ethnic communities in Southwest China, the region with the highest minority ethnic population, the most complex ethnic composition, and the richest and most diverse ethnic culture, mainly in Yunnan, Guizhou, Sichuan, and Chongqing. Specifically, according to the Chinese sixth population census data (Chinese National Bureau of Statistics, 2010), there are 25 minority ethnic groups who have lived for generations in Yunnan Province alone, accounting for 13.71% of the total minority ethnic population in the country. To ensure minority ethnic diversity, several *Minzu* schools, mainly colleges and universities, in Southwest China, were determined as the sampling units. The survey was conducted with the help of school administrators who forwarded our online survey to classes with a more balanced gender ratio of minority ethnic students. It took participants approximately 20 min to complete the set of scales and demographic questions, and they were allowed to pause the surveys and return them within 3 days. We paid 10 Yuan RMB for each completed survey. A total of 1398 surveys were completed, and the effective survey rate was 93.6%.

### Results and Discussion

#### Reliability of the CMEVQ and CMEVEBQ

We dealt with the reverse scoring items in the CSE, and computed the mean scores for each of the subscales of all questionnaires, except for the IIS. As shown in Tables 3, 4, the internal reliability of each subscale with three items in the CMEVQ and CMEVEBQ was acceptable. Reliability coefficients of the total scores were 0.814 for CMEVQ and 0.805 for CMEVEBQ. Despite the internal consistency of several factors being somewhat below the desired level, they were acceptable for subscales with fewer than five items apiece (Nunnally and Bernstein, 1994).

#### Validity of the CMEVQ and CMEVEBQ

##### Structure validity

We rechecked the goodness of fit of the two models in Study 1. Prior to analysis, we replaced the missing data, constituting less than 1% in the CMEVEBQ, with Bayesian interpolation (Meng, 1994). The indexes were used to evaluate the goodness of fit as follows: χ^2^/*df* ratios (i.e., lower than 3; Kline, 2015), the root mean square error of approximation (RMSEA) values (i.e., lower than 0.06; Thompson, 2004), the comparative fit index (CFI), the goodness of fit index (GFI) and adjusted-goodness-of-fit index (AGI; i.e., higher than 0.90; Bentler, 1990), and the standardized root mean square residual (SRMR) values (i.e., lower than 0.06; Hu and Bentler, 1999). Our results for the CMEVQ and CMEVEBQ are shown in line 2 of the two panels of Table 5. All indices were better except for the Norm Chi-square for the CMEVQ, which was higher than desired but still acceptable at a critical level of 5 (Lomax and Schumacker, 2004).

The fit indexes of the four-factor model for the CMEVQ and CMEVEBQ are presented in line 4 of the two panels of Table 5. Some indexes were lower than those of the six-factor model, but most were acceptable. This suggested that the four-factor models of the two newly developed questionnaires were also applicable in practice. The specific items for each factor are included in the section “Implications of Using the CMEVQ and CMEVEBQ,” for use in future studies.

##### Criterion-related validity

We conducted Pearson correlations between the existing ethnic variables with equivalent or overlapping conceptual content and 12 new variables to examine the criterion validity. As shown in Table 6, each subscale of the CMEVQ and CMEVEBQ was significantly positively related to all of the criterion variables, except for two MEA subscales, which were negatively related to them. The inter-factor correlations in the CMEVQ ranged from 0.35 to 0.74 and the average correlation was 0.56, which was significantly higher than the average correlation of the five ethnic value factors with the IIS of 0.27, Social Norm of 0.21, Preference for Ethnic Things of 0.41, Ethnic Acceptance of 0.37, ESC of 0.38, Membership Esteem of 0.31, Private Collective Self-Esteem of 0.35, and Importance to Identity of 0.40, χ^2^(8) = 290.93, *p* < 0.001, ρ = 0.350 (Edwards, 1984). The inter-factor correlations in the CMEVEBQ ranged from 0.20 to 0.69 with an average correlation of 0.48, which was also significantly higher than the average correlation of five ethnic behavior factors with the criterion variables described above by 0.27, 0.20, 0.41, 0.32, 0.34, 0.31, 0.30, and 0.38, respectively, χ^2^(8) = 204.9, *p* < 0.001, ρ = 0.305. This indicated that the subscales of the two minority ethnic questionnaires have observable convergent and discriminant validity. Furthermore, the factor Preference for Ethnic Things presented a stronger correlation with more subscales in the CMEVQ and CMEVEBQ.

**TABLE 6:** Means and standard deviations of the subscales, and Pearson correlations among the scales in Study 2.

	***M***	***SD***	**Correlations among the variables**											
			
			**VMEC**	**VMEE**	**VMEIV**	**VMEA**	**VMEIH**	**VMEM**	**BMEC**	**BMEE**	**BMEIV**	**BMEA**	**BMEIH**	**BMEM**
Value in MEC	4.09	0.92	—	0.70^∗∗∗^	0.70^∗∗∗^	–0.35^∗∗∗^	0.56^∗∗∗^	0.52^∗∗∗^						
Value in MEE	4.74	0.88		—	0.73^∗∗∗^	–0.45^∗∗∗^	0.74^∗∗∗^	0.60^∗∗∗^						
Value in MEIV	4.14	1.07			—	–0.39^∗∗∗^	0.64^∗∗∗^	0.50^∗∗∗^						
Value in MEA	2.55	1.08				—	–0.48^∗∗∗^	–0.35^∗∗∗^						
Value in MEIH	4.97	0.75					—	0.71^∗∗∗^						
Value in MEM	4.93	0.81						—						
Behavior in MEC	2.50	0.90	0.48^∗∗∗^	0.52^∗∗∗^	0.54^∗∗^	–0.28^∗∗∗^	0.40^∗∗∗^	0.30^∗∗∗^	—	0.66^∗∗∗^	0.56^∗∗∗^	–0.38^∗∗∗^	0.50^∗∗∗^	0.41^∗∗∗^
Behavior in MEE	2.28	0.80	0.45^∗∗∗^	0.55^∗∗^	0.52^∗∗∗^	–0.34^∗∗∗^	0.45^∗∗∗^	0.34^∗∗∗^		—	0.69^∗∗∗^	–0.41^∗∗∗^	0.54^∗∗∗^	0.44^∗∗∗^
Behavior in MEIV	3.06	0.69	0.44^∗∗∗^	0.56^∗∗^	0.53^∗∗∗^	–0.39^∗∗∗^	0.55^∗∗^	0.43^∗∗^			—	–0.44^∗∗∗^	0.56^∗∗∗^	0.46^∗∗∗^
Behavior in MEA	0.91	0.78	–0.38^∗∗∗^	–0.45^∗∗∗^	–0.40^∗∗∗^	0.43^∗∗∗^	–0.41^∗∗∗^	–0.32^∗∗∗^				—	–0.32^∗∗∗^	–0.20^∗∗∗^
Behavior in MEIH	2.92	0.86	0.36^∗∗∗^	0.42^∗∗∗^	0.37^∗∗∗^	–0.28^∗∗∗^	0.44^∗∗^	0.35^∗∗∗^					—	0.64^∗∗∗^
Behavior in MEM	2.81	0.68	0.26^∗∗∗^	0.31^∗∗∗^	0.27^∗∗∗^	–0.18^∗∗∗^	0.33^∗∗∗^	0.26^∗∗∗^						—
IIS	4.56	1.55	0.27^∗∗∗^	0.34^∗∗∗^	0.21^∗∗∗^	–0.24^∗∗∗^	0.31^∗∗∗^	0.23^∗∗∗^	0.41^∗∗∗^	0.32^∗∗∗^	0.28^∗∗∗^	–0.34^∗∗∗^	0.20^∗∗∗^	0.14^∗∗∗^
CMACIQ social norm	4.62	0.50	0.18^∗∗∗^	0.23^∗∗∗^	0.12^∗∗^	–0.14^∗∗∗^	0.27^∗∗^	0.24^∗∗∗^	0.12^∗∗^	0.13^∗∗^	0.24^∗∗∗^	–0.21^∗∗∗^	0.25^∗∗∗^	0.24^∗∗∗^
CMACIQ preference for ethnic things	4.19	0.60	0.39^∗∗∗^	0.53^∗∗∗^	0.38^∗∗∗^	–0.24^∗∗∗^	0.48^∗∗∗^	0.29^∗∗∗^	0.49^∗∗∗^	0.48^∗∗∗^	0.44^∗∗∗^	–0.43^∗∗∗^	0.34^∗∗∗^	0.28^∗∗∗^
CMACIQ ethnic acceptance	4.17	0.56	0.34^∗∗∗^	0.45^∗∗∗^	0.35^∗∗∗^	–0.17^∗∗∗^	0.43^∗∗∗^	0.30^∗∗∗^	0.36^∗∗∗^	0.38^∗∗∗^	0.37^∗∗∗^	–0.35^∗∗∗^	0.29^∗∗∗^	0.21^∗∗∗^
Ethnic Self-Categorization	3.47	0.77	0.44^∗∗∗^	0.45^∗∗∗^	0.37^∗∗∗^	–0.21^∗∗∗^	0.37^∗∗∗^	0.27^∗∗∗^	0.53^∗∗∗^	0.46^∗∗∗^	.28^∗∗∗^	–0.38^∗∗∗^	0.24^∗∗∗^	0.16^∗∗∗^
CSE in membership	5.20	1.00	0.27^∗∗∗^	0.44^∗∗∗^	0.23^∗∗∗^	–0.24^∗∗∗^	0.33^∗∗∗^	0.27^∗∗∗^	0.35^∗∗∗^	0.37^∗∗∗^	0.26^∗∗∗^	–0.37^∗∗∗^	0.28^∗∗∗^	0.28^∗∗∗^
CSE in private	6.22	0.73	0.27^∗∗∗^	0.41^∗∗∗^	0.27^∗∗∗^	–0.256^∗∗∗^	0.44^∗∗∗^	0.38^∗∗∗^	0.26^∗∗∗^	0.29^∗∗∗^	0.41^∗∗∗^	–0.47^∗∗∗^	0.30^∗∗∗^	0.23^∗∗∗^
CSE importance to identity	5.34	1.06	0.43^∗∗∗^	0.47^∗∗∗^	0.36^∗∗∗^	–0.32^∗∗∗^	0.47^∗∗∗^	0.30^∗∗∗^	0.45^∗∗∗^	0.44^∗∗∗^	0.34^∗∗∗^	–0.46^∗∗∗^	0.29^∗∗∗^	0.23^∗∗∗^
Gender (1 = male, 0 = female)			–0.01	0.02	–0.01	–0.08^∗∗^	0.02	0.02	–0.04	–0.05	0.04	–0.01	–0.05	–0.09^∗∗^
Parental ethnicity (1 = same, 0 = different)			–0.14	–0.12^∗∗∗^	–0.14^∗∗∗^	0.04	–0.09^∗∗^	−0.07^∗^	–0.19^∗∗∗^	–0.04	–0.09^∗∗^	0.09^∗∗^	−0.06^∗^	–0.02
Residence status (1 = gathered-residing, 0 = scattered-residing)			–0.11^∗∗∗^	–0.11^∗∗∗^	–0.15^∗∗∗^	0.06*	–0.08^∗∗^	−0.06^∗^	–0.18^∗∗∗^	–0.08^∗∗^	–0.11^∗∗∗^	0.05	−0.07^∗^	–0.03
Source area (1 = urban, 0 = rural)			0.04	0.10^∗∗∗^	0.10^∗∗∗^	–0.08^∗∗^	0.06^∗^	0.03	0.08^∗∗^	0.05	0.06^∗^	−0.06^∗^	0.03	−0.06^∗^
Religious affiliation (1 = no, 0 = yes)			0.21^∗∗∗^	0.14^∗∗∗^	0.21^∗∗∗^	–0.05	0.06^∗^	0.02	0.31^∗∗∗^	0.13^∗∗∗^	0.07^∗^	–0.13^∗∗∗^	0.05	0.08^∗∗^

*^∗^*p* < 0.05; ^∗∗^*p* < 0.01; ^∗∗∗^*p* < 0.001. CMACIQ, Chinese Multiethnic Adolescent Cultural Identity Questionnaire; CSE, Collective Self-Esteem Scale; IIS, Inclusion of In-group in the Self. The initial letter V of the subscale name means “in value questionnaire,” and B means “in behavior questionnaire.” MEC, Minority Ethnic Consciousness; MEE, Minority Ethnic Exploration; MEIV, Minority Ethnic Involvement; MEA, Minority Ethnic Alienation; MEIH, Minority Ethnic Inheritance; MEM, Minority Ethnic Mastery.*

##### Discriminant validity

We further examined the discriminant validity of the two questionnaires with a K-means cluster analysis, using IIS rating as a clustering variable. The participants were divided into three clusters according to the distances between the final cluster centers of 1.78 and 2.26. We conducted an independent sample *t*-test between the alienation group (*n* = 190) and closeness group (*n* = 638), and the former (*M*s = 1.51−4.49, *SD*s = 0.80−1.16) scored significantly lower on five dimensions of the values and behavior scales (all except MEA) than the latter group (*M*s = 2.35 − 5.02, *SD*s = 0.70 −0.99), *t*s = 6.29 − 15.16, *p* < 0.001; however, the alienation group scored significantly higher on the MEA dimension (*M*_v__–__m__e__a_ = 2.92 and *M*_b–__m__e__a_ = 1.55, *SD*_v__–__m__e__a_ = 1.04 and *SD*_b__–__m__e__a_ = 0.92) than the closeness group (*M*_v__–__m__e__a_ = 2.31 and *M*_b__–__m__e__a_ = 0.92, *SD*_v__–__m__e__a_ = 0.99 and *SD*_b__–__m__e__a_ = 0.74), *t*s = 7.43 and 9.62, *p* < 0.001. This indicated that there was a good discriminant validity in the CMEVQ and CMEVEBQ.

##### Ecological validity

The results suggest that stronger ethnicity may exist in minority ethnic participants whose parents were of the same minority ethnic group, who were from minority-inhabited areas, or who were born in rural areas (e.g., Deng and Zhang, 2014; Ji and Li, 2016), while ethnicity may differ by gender and religious affiliation (Tu, 2010). Based on the significant correlations between demographic variables and subscales in the CMEVQ and CMEVEBQ (see Table 6), we examined differences using the independent sample *t*-test (see Table 7).

**TABLE 7:** Independent samples test of demographic variables among the subscales of CEMVQ and CEMVEBQ in Study 2.

**DV**	**Subscale**	***M***	***SD***	***t***	**DV**	**Subscale**	***M***	***SD***	***t***
Parental ethnicity	VMEC	4.18	0.91	4.90^∗∗∗^	Gender	VMEA	2.63	1.08	2.87^∗∗^
		3.91	1.02		1 = male (*n* = 521)		2.46	0.97	
1 = same (*n* = 805)	VMEE	4.50	0.92	4.10^∗∗∗^	2 = female (*n* = 788)	BMEM	2.76	0.74	3.36^∗∗^
		4.27	1.00				2.62	0.73	
2 = different (*n* = 504)	VMEIV	4.16	0.97	4.83^∗∗∗^		VMEE	4.32	0.97	3.42^∗∗^
		3.88	1.09		Source area		4.54	0.93	
	VMEIH	4.83	0.85	3.13^∗∗^	1 = urban (*n* = 378)	VMEIV	3.96	1.04	3.40^∗∗^
		4.67	0.90				4.19	0.99	
	VMEM	4.90	0.90	2.49^∗^	2 = rural (*n* = 548)	VMEA	2.59	1.01	2.76^∗∗^
		4.78	0.88				2.40	0.99	
	BMEC	2.40	0.81	6.85^∗∗∗^		VMEIH	4.72	0.88	2.10^∗^
		2.06	0.92				4.84	0.87	
	BMEIV	2.73	0.76	3.23^∗∗^		BMEC	2.21	0.92	2.65^∗∗^
		2.58	0.83				2.37	0.81	
	BMEA	1.04	0.77	3.30^∗∗^		BMEIV	2.63	0.85	1.99^∗^
		1.20	0.87				2.73	0.73	
	BMEIH	2.69	0.86	2.04^∗^		BMEA	1.16	0.86	2.01^∗^
		2.59	0.90				1.05	0.79	
Residence status	VMEC	4.16	0.92	4.08^∗∗∗^		BMEM	2.72	0.75	2.25^∗^
		3.94	1.01				2.61	0.71	
1 = gathered-residing (*n* = 809)	VMEE	4.49	0.93	3.99^∗∗∗^	Religious affiliation	VMEC	3.96	0.97	8.40^∗∗∗^
		4.28	0.98				4.42	0.84	
	VMEIV	4.17	1.01	5.34^∗∗∗^	1 = no (*n* = 974)	VMEE	4.33	0.97	5.48^∗∗∗^
2 = scattered-residing (*n* = 500)		3.86	1.02				4.64	0.86	
	VMEA	2.48	1.02	2.18^∗^	2 = yes (*n* = 325)	VMEIV	3.93	1.03	7.91^∗∗∗^
		2.61	1.01				4.41	0.94	
	VMEIH	4.83	0.85	3.04^∗∗^		VMEA	2.56	1.02	1.85
		4.68	0.90				2.44	1.02	(0.065)
	VMEM	4.90	0.88	2.30^∗^		VMEIH	4.74	0.88	1.97^∗^
		4.78	0.90				4.85	0.84	
	BMEC	2.40	0.83	6.73^∗∗∗^		BMEC	2.11	0.85	12.52^∗∗∗^
		2.07	0.89				2.73	0.75	
	BMEE	2.16	0.83	2.84^∗∗^		BMEE	2.05	0.84	4.70^∗∗∗^
		2.03	0.87				2.30	0.84	
	BMEIV	2.74	0.74	3.81^∗∗∗^		BMEIV	2.64	0.81	2.45^∗^
		2.56	0.85				2.75	0.71	
	BMEA	1.07	0.81	1.91 (0.057)		BMEA	1.17	0.82	4.81^∗∗∗^
		1.16	0.81				0.92	0.76	
	BMEIH	2.70	0.84	2.29^∗^		BMEM	2.64	0.76	2.97^∗∗^
		2.58	0.93				2.77	0.68	

*^∗^*p* < 0.05; ^∗∗^*p* < 0.01; ^∗∗∗^*p* < 0.001. DV, Demographic variable. The initial letter V of the subscale name means “in value questionnaire,” and B means “in behavior questionnaire.” MEC, Minority Ethnic Consciousness; MEE, Minority Ethnic Exploration; MEIV, Minority Ethnic Involvement; MEA, Minority Ethnic Alienation; MEIH, Minority Ethnic Inheritance; MEM, Minority Ethnic Mastery.*

In terms of gender, men scored higher than women on the V-MEA and B-MEM. Generally, men tended to leave their families and original ethnic settlements to find employment, and they had access to more external cultures than their own; women, on the other hand, were more likely to stay at home and to stay in contact with and inherit more indigenous ethnic culture (Phinney, 1990). This may lead to men gradually leaving behind ethnic indigenous context and weakening their ethnic consciousness. Men also tended to think that it was valuable to have good relationships with other ethnic groups (Tu, 2010, p. 20), reflected by MEM.

Parental ethnicity and family residence area (i.e., gathered or scattered, rural or urban) may also influence minority ethnicity (Phinney, 1990; Wan and Wang, 2004; Chen and Chen, 2018). Specifically, there may be stronger ethnicity for ethnic minorities living in rural, ethnic gathered-residence areas, and for families with parents of the same ethnic group, than those living in urban, ethnic scattered-residence areas, and families with parents of different ethnic groups. Parents of the same minority speak the same language, have common customs, and attend shared festivals with their children wearing ethnic clothing. These behaviors as ethnic symbols usually occur more diversely and frequently in minority gathered-residence areas, so ethnicity in such a context is strengthened and passed down from generation to generation. By comparison, parents of different ethnic groups would choose whether or not to follow their own ethnic customs according to the needs of the family, which was also applicable to minority dispersed-residence areas represented by mainstream culture or multicultural values. Ethnic minorities living in dispersed-residence areas perceive the possibility of engaging in ethnic behaviors by a small number of their own ethnic members including themselves in a non-indigenous ethnic context, based on the available resources and opportunities for such behaviors (Theory of Planned Behavior; Tesser and Shaffer, 1990). Furthermore, due to geographical remoteness and slow economic development, minority ethnic culture in rural areas was affected less by mainstream and external culture and remained relatively complete and unique; thus, minorities there have a higher ethnic identity than those living in more modern and multicultural urban areas (Ji and Li, 2016). Accordingly, a family with parents of the same ethnic group, a social context of ethnic gathered-residence, and a closed area away from multicultural influences may be factors that promote enhanced ethnicity. Interestingly, the developed subscales of the CMEVQ and CMEVEBQ presented significant differences in the three demographic variables, which supported the few existing conclusions and provided more accurate descriptions of ethnic-minority-values and behaviors.

Regarding religious affiliation, only 25.6% (*n* = 335) of respondents reported being religious believers. General speaking, the term “religious believer” refers to those who identify with one of the world’s top three religions, i.e., Islam, Christianity and Buddhism (as reported by interviews in the pilot study). In fact, natural religion exists widely among the ethnic minorities of Southwest China, the source of our study sample, and is closely integrated into the daily lives of minority ethnic people. Furthermore, these ethnic groups still tend to personify natural forces and frequently hold various related activities (Lu, 2006). It may be said that our minority ethnic participants who adhere to the tenets of Islam, Christianity or Buddhism also remain faithful to the natural religions passed down by their own ethnic ancestors. The two types of belief coexist in the same minority ethnic group or even the same ethnic member, and continue for a long time. In determining how ethnicity is presented in such cases, our results found that despite fewer participants reporting to be religious believers, believers scored higher than non-believers on 9 out of 10 positive dimensions, both for ethnic-minority-values and value-expressive behaviors. Thus, religion may contribute to ethnic identity and enhance minority ethnic ethnicity.

As we expected, participants who scored high on the MEC, MEE, MEIV, MEIH, and MEM subscales scored low on the MEA subscale, and MEA scored higher on demographic factors that have been suggested to weaken ethnicity, compared to those that may promote ethnicity. These findings also confirmed the construct and external validity of the two newly developed questionnaires and increased our confidence in using this dimension alone to check the ethnicity of ethnic members.

#### Associations Between Values and Value-Expressive Behaviors of Ethnic Minorities

Regarding ethnic-minority-value–behavior correlations, as shown in Table 6, all Pearson correlations on the diagonal axis were significant. Two (MEE and MEIH) of six minority ethnic-minority-values correlated most strongly with their corresponding ethnic-minority-behaviors than with the other five sets of behavior. MEC, MEIV, and MEA value correlated more strongly with its postulated behaviors than with the other five behavior sets. Only MEM value had a low correlation with its postulated behaviors set (*r* = 0.26).

Table 6 also shows that the MEA dimension is significantly negatively correlated with five other facets both in the CMEVQ and CMEVEBQ. This suggests that minority ethnic individuals who are psychologically or practically distant from their own minority ethnic groups have lower self-perception as members of those groups, seldom seek opportunities to explore their ethnic knowledge subjectively, and are indifferent as to care whether their own ethnicity is passed down through the generations. This supports the result in Wan and Wang (2004) that negative ethnic identity was positively correlated with cultural separation of groups. Moreover, such ethnic members themselves have been almost integrated into mainstream culture, as well having no self-perceived differences between their own ethnic group and others, which was also what the MEM facet meant. Thus, MEA has all the lowest correlations with MEM within value facets, behavior facets, and between two value-behavior correlations: −0.35, −0.20, −0.18, and −0.32, respectively.

To further verify their relationship, we also checked the predictive roles of ethnic values to its corresponding ethnic behaviors (see Table 8). Separately, with each of the six behavior subscales as the target variable, the predictors entered in the multiple regression comprised the six value subscales. Four of the six predictors, that is MEE, MEIV, MEA, and MEIH, explained the most variance of its postulated behavior and this added new statistical evidence that value is correlated most strongly with behavior in value-consistent directions, just as Schwartz and Butenko (2014) validated in the Russian context and Schwartz et al. (2017) in four countries. In contrast, with the behavior in minority ethnic consciousness (B-MEC) as the target variable, its most variance (β = 0.30) was explained by the predictor of value in minority ethnic involvement (V-MEIV) rather than its postulated ethnic value, which was consistent with the results of Pearson correlations, demonstrating that B-MEC correlated more strongly with V-MEIV than with the other five ethnic values; this suggested that the higher the emotional involvement of ethnic minorities in their own ethnic groups, the more strongly they self-perceived their ethnicity in daily behavior. This is consistent with the literature stating that respondents with higher IIS scores tended to report that their behaviors are more influenced by ingroup members than those with lower IIS scores (Tropp and Wright, 2001). Another potential coincidence was that the IIS correlated most strongly with B-MEC in this study.

**TABLE 8:** Regression of ethnic minority value factors on minority value-expression behavior (only predictive independent variables that are significant or marginally significant).

**DV**	**IV**	**USC**		**SC**	***t***	**CS**		**DW (U)**	***R*^2^**
							
		***B***	***SE***	**β**		**Tolerance**	**VIF**		
B-MEC	(Constant)	0.27	0.17		1.62 (0.106)			1.90	0.34
	V-MEC	0.13	0.03	0.14	4.05^∗∗∗^	0.43	2.35		
	V-MEE	0.21	0.04	0.23	5.61^∗∗∗^	0.30	3.36		
	V-MEIV	0.25	0.03	0.30	8.18^∗∗∗^	0.38	2.64		
	V-MEM	–0.06	0.03	–0.06	1.91 (0.056)	0.47	2.13		
B-MEE	(Constant)	0.21	0.16		1.31 (0.189)			1.95	0.34
	V-MEE	0.28	0.04	0.31	7.56^∗∗∗^	0.30	3.36		
	V-MEIV	0.18	0.03	0.21	5.87^∗∗∗^	0.38	2.64		
	V-MEA	–0.08	0.02	–0.10	3.68^∗∗∗^	0.74	1.34		
B-MEIV	(Constant)	0.55	0.15		3.72^∗∗∗^			2.06	0.38
	V-MEE	0.16	0.03	0.19	4.84^∗∗∗^	0.30	3.36		
	V-MEIV	0.16	0.03	0.21	5.80^∗∗∗^	0.38	2.64		
	V-MEA	–0.09	0.02	–0.11	4.53^∗∗∗^	0.74	1.34		
	V-MEIH	0.17	0.04	0.19	4.87^∗∗∗^	0.31	3.18		
B-MEA	(Constant)	2.02	0.16		12.26^∗∗∗^			1.95	0.28
	V-MEC	–0.06	0.03	–0.07	1.91 (0.056)	0.43	2.35		
	V-MEE	–0.17	0.04	–0.19	4.50^∗∗∗^	0.30	3.36		
	V-MEIV	–0.06	0.03	–0.08	2.07^∗^	0.38	2.64		
	V-MEA	0.21	0.02	0.26	9.51^∗∗∗^	0.74	1.34		
B-MEIH	(Constant)	0.59	0.18		3.20^∗∗^			1.91	0.22
	V-MEC	0.07	0.03	0.07	1.98^∗^	0.43	2.35		
	V-MEE	0.10	0.04	0.11	2.44^∗^	0.30	3.36		
	V-MEA	–0.05	0.02	–0.06	2.07^∗^	0.74	1.34		
	V-MEIH	0.22	0.04	0.22	5.05^∗∗∗^	0.31	3.18		
B-MEM	(Constant)	1.22	0.16		7.40^∗∗∗^			1.88	0.12
	V-MEE	0.07	0.04	0.09	1.89 (0.059)	0.30	3.36		
	V-MEIH	0.17	0.04	0.20	4.38^∗∗∗^	0.31	3.18		

*^∗^*p* < 0.05; ^∗∗^*p* < 0.01; ^∗∗∗^*p* < 0.001. DV, Dependent variable; IV, Independent variable; USC, Unstandardized coefficients; SC, Standardized coefficients; CS, Collinearity statistics; DW, Durbin-Watson. The initial letter V of the subscale name means “in value questionnaire,” and B means “in behavior questionnaire.” MEC, Minority Ethnic Consciousness; MEE, Minority Ethnic Exploration; MEIV, Minority Ethnic Involvement; MEA, Minority Ethnic Alienation; MEIH, Minority Ethnic Inheritance; MEM, Minority Ethnic Mastery.*

Most ethnic values may predict ethnic behaviors either together or separately, suggesting that the two new questionnaires are well-matched and can even be mutually validated variables. However, the values MEM did not present the expected strong correlation with its postulated MEM behavior (*r* = 0.26, *p* < 0.001); there was no predictive role for the former on the latter, and the internal consistency of V-MEM was particularly low. There may be several reasons for this. Mastery as a cultural value orientation is the correlated polar dimension of harmony. In contrast to harmony, which emphasizes fitting into the social and natural world or acceptance rather than change, mastery encourages active self-assertion to attain group or personal goals (Schwartz, 2014). Ethnicity for minority ethnic groups requires changing to adapt to social development in order to achieve ethnic inheritance, rather than fitting into it (Verkuyten, 2005). The MEM dimension in this study aimed to assess how to deal with the relationship between a minority ethnic group and the external world through ethnic values and value-expressive behaviors. However, ethnic mastery is difficult to represent with only a few value items and typical behavioral instantiation, as the concept is too broad (e.g., Maio, 2010; Hanel et al., 2018). Furthermore, the harmonious and reciprocal relations among Chinese ethnic groups have not positioned this as a necessary issue among minority ethnic populations, suggesting that this needs to be examined in more empirical ethnic studies. Another reason may be poor content representation of the MEM facet. Individuals can have a strong sense of belonging to a group and yet not necessarily be engaged in daily ethnic activities (Phinney and Ong, 2007). Finally, individuals and groups have different value priorities or hierarchies, and will differ in their use of the response scales, especially among cross-cultural samples (Schwartz, 2006). This is also a possible explanation for the low alpha reliabilities obtained in the current study, as the participants in the Study 2 represented nearly 40 Chinese ethnic minorities. The low reliabilities of values scales also appeared in Schwartz’s values researches (e.g., Schwartz et al., 2001; Devos et al., 2002; Schwartz and Rubel, 2005).

### Limitations and Future Directions

Several limitations to the current research need to be noted, and addressed in future studies. First, our participants required at least primary education to complete this survey independently, and this excluded some ethnic minorities who have poor education levels but may have many contacts with their ethnic cultures and have strong ethnicity. In addition, this study was mainly limited to college students without consideration of age and professional factors; thus, future studies should examine the ethnic values and ethnic behavior model and their metric properties in other minority ethnic populations. Second, in view of smaller samples for particular groups or totally unequal population ratio for diverse groups, the validity of the new questionnaires cannot be verified between minority ethnic groups. In future work, we plan to adopt a stratified sampling procedure to predetermine the size of each minority ethnic group to try to balance the population number between groups. Third, the current study investigated the statistical relationship between ethnic values and its corresponding behaviors. In future studies, it is necessary to test its causal relationship through the priming approach. Fourth, B-MEM had a lower internal consistency in newly developed scales than the generally agreed criteria, and several higher inter-factor correlations appeared in the CMEVQ. In view of the need for the external validity of the two newly developed scales, we retained the factors to further explore their content in future studies. Finally, this survey was limited by its exclusive use of self-reported data, which may have been affected by individuals’ incomplete memory and self-identification, and is also motivated by consistency seeking or social desirability. Rating from other observers on ethnic-minority-values and value-expressive behaviors may further our understanding of this domain; therefore, future studies may build the relation of self-rating to other-rating to reduce the response bias of participants as much as possible.

## Conclusion

Based on Verkuyten’s (2005) four “ways of ethnicity,” value theory, and an operational definition of ethnic-minority-values, we developed the CMEVQ and CMEVEBQ to systematically examine the psychological structure of ethnic-minority-values and value-expressive behavior in the Chinese multiethnic population. CMEVEBQ as a behavioral measure was generated specifically to match the value dimensions. The two new questionnaires from the ethnic “inside” measured “being,” “feeling,” “knowing,” and “doing” with minority ethnic consciousness, exploration, involvement, alienation, inheritance, and mastery. The questionnaires advance previous measures by separately including the values and behavioral descriptions expressing the importance of ethnicity as covered by their six shared subscales within two focused and practical instruments of 18 items each.

In Study 1, the results of EFA, SEM, and MG-CFA indicated that the ethnic-minority-value and value-expressive behavior models were robust and had reasonable psychometric properties across ethnic settlement regions. In Study 2, the relevance examination with criterion scales, K-means cluster analysis, and difference tests across demographic factors offered further support for the convergent, divergent, and ecological validity of the CMEVQ and CMEVEBQ. The results of associations between ethnic-minority-values and value-expressive behavior by Pearson correlation and multiple regression provided further statistical and sample evidence that values serves as a guide of behavioral tendencies. In addition, EMA, as an assumed factor of cognitive unimportance in ethnicity, was negatively correlated with all variables of cognitive importance in ethnicity in this study, which enhanced the credibility of the other five shared factors in measuring ethnic-minority-values and their corresponding behaviors. Therefore, the ethnicity of a minority ethnic member is a significant component of their overall self-construal, and they can automatically categorize themselves and others into different groups in terms of ethnicity. In conclusion, the current research provides statistical support and a method to practically measure the values and value-expressive behaviors of ethnic minorities, as well as develops a new path to represent the importance of ethnicity for ethnic minorities and extends the research field on value theory. All of these have contributed to passing down the cultural uniqueness of ethnic minorities and to further in-depth research by researchers and practitioners in the ethnological field.

## Implications of Using the CMEVQ and CMEVEBQ

The current status of the CMEVQ and CMEVEBQ suggests that these measures can be useful tools to facilitate understanding of the importance of ethnicity by ethnic-minority-values and value-expressive behaviors in broad strokes. The two questionnaires can be used alone or together. Our empirical findings on the shared six-factor structure, as well as the interpretability of specific factor scores, indicate that the measures captured a basic understanding of ethnic-minority-value and four “ways of ethnicity” in Chinese culture. Further, the two sets of items were sufficiently heterogeneous to cover the importance and common contents of minority ethnic cultures and behavioral manifestations.

As values and behaviors are concrete concepts that involve specific content, such as tradition, achievement, or power (Schwartz, 2014; Schwartz et al., 2017), rather than a general or common concept that can be presented by the overall mean scores for all subscales, it would be appropriate and meaningful to compute the mean subscale scores. These subscale scores could be used together in the same analyses to represent diverse domains of importance of ethnicity for ethnic minorities.

As for the four-factor model, we named the four factors *Ethnic Being* (EB), *Ethnic Feeling* (EF), *Ethnic Knowing* (EK), and *Ethnic Doing* (ED), to distinguish them from those of the six-factor model for both questionnaires. For the CMEVQ in Table 3, Values-EB includes MEIV1-3 and MEC1-2 (5 items); Values-EF includes MEA1-3 (3 items); Values-EK includes MEM1-3 (3 items); and Values-ED includes MEE1-3, MEIH1-3 and MEC3 (7 items). For the CMEVEBQ in Table 4, Behavior-EB includes MEC1-3 and MEE1-3 (6 items); Behavior-EF includes MEIV1-3 (3 items); Behavior-EK includes MEA1-3 (3 items); and Behavior-ED includes MEIH-3 and MEM1-3 (6 items).

## Attachment

The item content of the newly developed questionnaires in this study are shown in Tables 3, 4, respectively. Their instructions are as follows:

### Instruction on the CMEVQ

Here, we briefly describe different people. Please read the following statements carefully and consider how much the person described is or is not like you. Rate the extent to which the person described is like you, using the following scale: 1 (*not like me*) to 6 (*very much like me*).

### Instruction for the CMEVEBQ

Here, we briefly describe different ethnic behaviors. Please read the following statements carefully and rate the behavior that best describes how often you have engaged in the behavior relative to your opportunities to do so, on the following scale: 0 (*never*) to 4 (*always*). If you think you have never had even one opportunity to do the described behavior, mark the behavior with an *X*.

## Data Availability Statement

The datasets generated for this study are available on request to the corresponding author.

## Ethics Statement

All studies in this article were recommended and approved by the Ethics Committee of Southwest University. All participants were informed of the survey purpose and told that their responses were voluntary, confidential, and for research purposes only. They provided online informed consent in accordance with the Declaration of Helsinki. After completion, participants were paid for their participation and invited to contact the researcher if they had any further questions about the study.

## Author Contributions

YY designed the study, collected and analyzed the data, and drafted the initial manuscript. FL participated in the design of the questionnaires. FQ contributed to the data analyses. GJ and KY participated in issuing and collecting the questionnaires. YZ provided the critical comments and contributed to the revision. All authors approved the final version of the manuscript for submission.

## Conflict of Interest

The authors declare that the research was conducted in the absence of any commercial or financial relationships that could be construed as a potential conflict of interest.

## Footnotes

1 www.wenjuan.com
